# Integration of single-nuclei and spatial transcriptomics to decipher tumor phenotype predictive of relapse-free survival in Wilms tumor

**DOI:** 10.3389/fimmu.2025.1539897

**Published:** 2025-03-03

**Authors:** Ran Yang, Lulu Xie, Rui Wang, Yi Li, Yifei Lu, Baihui Liu, Shuyang Dai, Shan Zheng, Kuiran Dong, Rui Dong

**Affiliations:** ^1^ Department of Pediatric Surgery, Children’s Hospital of Fudan University, Shanghai Key Laboratory of Birth Defect, Shanghai, China; ^2^ Children’s Hospital of Fudan University (Xiamen Branch), Xiamen Children’s Hospital, Xiamen Key Laboratory of Pediatric General Surgery Diseases, Xiamen, China; ^3^ Shanghai Medical College, Fudan University, Shanghai, China

**Keywords:** Wilms tumor, recurrence, cancer stem cell, machine learning, immune microenvironment

## Abstract

**Background:**

Wilms tumor (WT) is the most common childhood renal malignancy, with recurrence linked to poor prognosis. Identifying the molecular features of tumor phenotypes that drive recurrence and discovering novel targets are crucial for improving treatment strategies and enhancing patient outcomes.

**Methods:**

Single-nuclei RNA sequencing (snRNA-seq), spatial transcriptomics (ST), bulk RNA-seq, and mutation/copy number data were curated from public databases. The Seurat package was used to process snRNA-seq and ST data. Scissor analysis was applied to identify tumor subpopulations associated with poor relapse-free survival (RFS). Univariate Cox and LASSO analyses were utilized to reduce features. A prognostic ensemble machine learning model was developed. Immunohistochemistry was used to validate the expression of key features in tumor tissues. The CellChat and Commot package was utilized to infer cellular interactions. The PERCEPTION computational pipeline was used to predict the response of tumor cells to chemotherapy and targeted therapies.

**Results:**

By integrating snRNA-seq and bulk RNA-seq data, we identified a subtype of Scissor+ tumor cells associated with poor RFS, predominantly derived from cap mesenchyme-like blastemal and fibroblast-like tumor subgroups. These cells displayed nephron progenitor signatures and cancer stem cell markers. A prognostic ensemble machine learning model was constructed based on the Scissor+ tumor signature to accurately predict patient RFS. TGFA was identified as the most significant feature in this model and validated by immunohistochemistry. Cellular communication analysis revealed strong associations between Scissor+ tumor cells and cancer-associated fibroblasts (CAFs) through IGF, SLIT, FGF, and PDGF pathways. ST data revealed that Scissor+ tumor cells were primarily located in immune-desert niche surrounded by CAFs. Despite reduced responsiveness to conventional chemotherapy, Scissor+ tumor cells were sensitive to EGFR inhibitors, providing insights into clinical intervention strategies for WT patients at high risk of recurrence.

**Conclusion:**

This study identified a relapse-associated tumor subtype resembling nephron progenitor cells, residing in immune-desert niches through interactions with CAFs. The proposed prognostic model could accurately identify patients at high risk of relapse, offering a promising method for clinical risk stratification. Targeting these cells with EGFR inhibitors, in combination with conventional chemotherapy, may provide a potential therapeutic strategy for WT patients.

## 1 Introduction

Wilms tumor (WT) is the most prevalent renal malignancy in infants and children, accounting for about 90% of pediatric renal tumors (1). The mean age at diagnosis is 44 months in patients with unilateral WT and 31 months in patients with bilateral WT (2, 3). As an embryonal tumor, WT is closely linked to early nephrogenesis and resembles fetal developing nephron cells (4).

Two major international collaborative groups, The Children’s Oncology Group Renal Tumor Committee(COG-RTC) (5) and the International Society of Pediatric Oncology Renal Tumor Study Group (SIOP-RTSG) (6) have refined the diagnostic and therapeutic guidelines which are used for the management of WT worldwide. Due to efficacious multidisciplinary therapy, the overall survival (OS) of patients with WT is high, reaching around 90% (7). However, disease recurrence or relapse still occurs in 20% patients within 2 years of diagnosis (7, 8), and the mortality rate is over 35% in patients with relapse (9, 10). Therefore, more comprehensive stratification criteria are required to accurately identify patients at high risk of recurrence and to refine personalized treatment strategies aimed at enhancing relapse-free survival (RFS).

Currently, both COG-RTC and SIOP-RTSG have utilized tumor stage, histological features, and tumor volume as critical prognostic indicators to stratify patients into distinct risk categories and to guide therapeutic interventions. Additionally, previous studies have underscored the significance of genetic aberrations in predicting increased risk of relapse and mortality, including mutations in genes such as *WT1, MYCN, TP53, IGF2, CTNNB1, SIX1/SIX2, AMER1*, and microRNA processing genes (11, 12), as well as copy number alterations such as gain of chromosome 1q and loss of heterozygosity (LOH) of chromosome 1p/16q/11p15 (11). Recent researchers have identified several gene signatures (13–15) closely related to the recurrence of WT, which may serve as predictors for RFS. Furthermore, the presence of cancer stem cells (CSCs) in WT has been established (16–18). However, the phenotype of tumor cells contributing to WT relapse has not been fully elucidated at single cell level. The tumor survival and growth are supported and furnished by tumor microenvironmental cells. The spatial relationships of relapse-associated tumor cells and the functional changes that occur within these spatial contexts to support WT recurrence are still not fully understood. Furthermore, the exploration of potential therapeutics targeting relapse-associated phenotypes has yet to be investigated.

Single-nucleus RNA sequencing (snRNA-seq) is a powerful technique for profiling cell types and investigating cellular heterogeneity by focusing on the capture and analysis of RNA from the nucleus. Compared to single-cell RNA sequencing (scRNA-seq), snRNA-seq offers significant advantages, particularly in efficiently measuring gene expression in individual cells derived from frozen or fixed tissues (19), as well as complex tissues (e.g., kidney (20), brain (21), heart (22)) that are challenging to dissociate. Spatial transcriptomics (ST), on the other hand, provides gene expression data while maintaining the spatial context of the cells (23). Integration of snRNA-seq with ST data enabled us gain a more comprehensive understanding of the underlying genetic and molecular mechanisms driving tumor biology (24).

**Figure 1:**
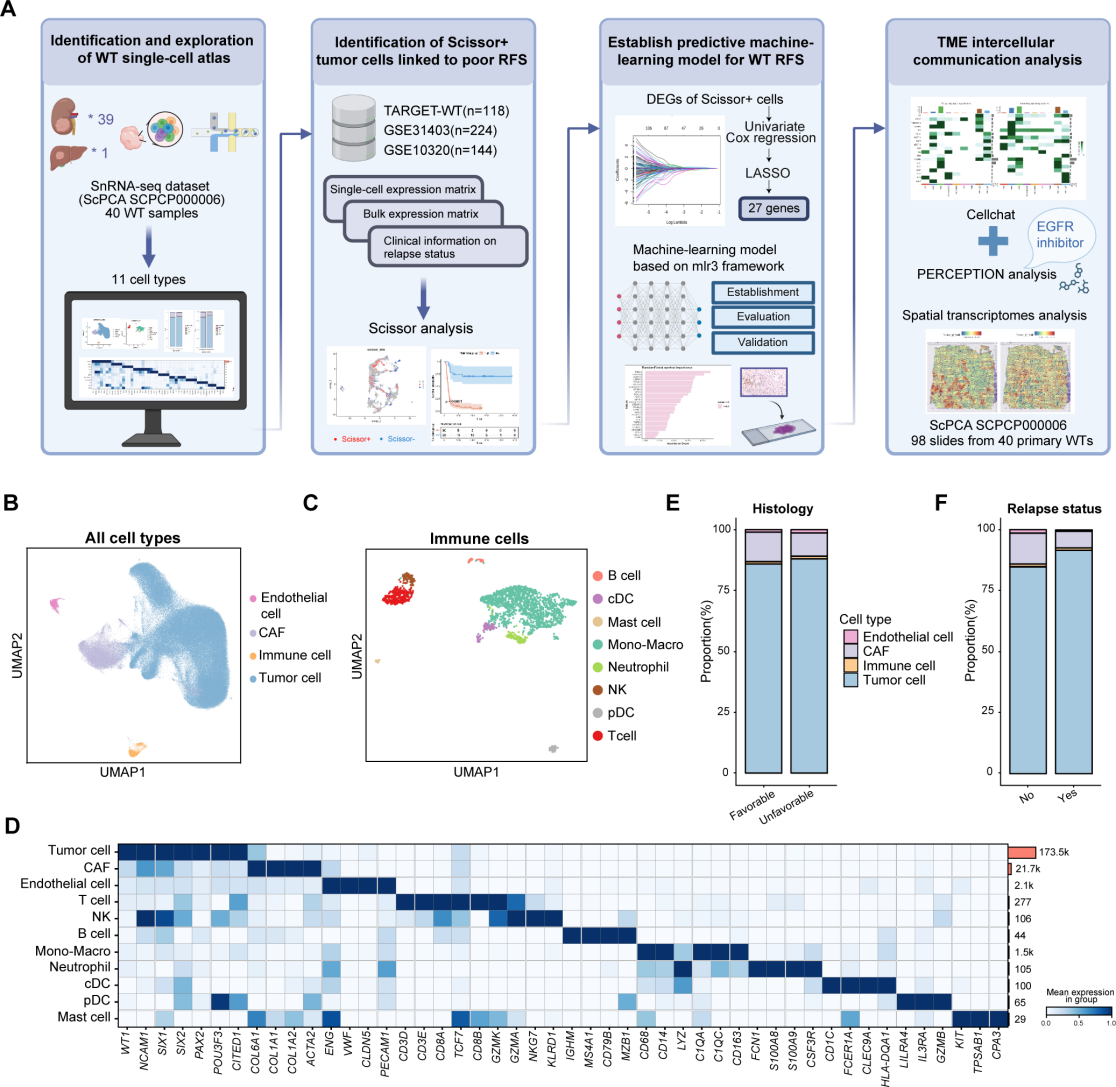
Overview of the study design and cell atlas from the published WT snRNA-seq dataset. **(A)** Overview of the study. Created by Biorender. **(B)** The UMAP visualization of all major cell types. **(C)** The UMAP visualization of immune cell types. **(D)** The heatmap illustrating the canonical cell markers across various cell types. **(E)** The bar plot showing the proportions of major cell types in unfavorable versus favorable samples. **(F)** The bar plot displaying the proportions of main cell types in samples with and without relapse after initial treatment. RFS, relapse-free survival; TME, tumor microenvironment; DEG, differentially-expressed gene; UMAP, uniform manifold approximation and projection; CAF, cancer-associated fibroblast; cDC, canonical dendritic cells; pDC, plasmacytoid dendritic cells.

In this study, we curated and integrated published snRNA-seq, bulk RNA sequencing, and ST data. Notably, we identified a subset of Scissor+ relapse-associated tumor cells which highly expressed nephron progenitor and CSC markers. A machine learning model based on this tumor signature predicts RFS and reflects genomic alterations tied to risk groups. Spatially, this tumor subset was encased by fibroblast stroma, restricting immune cell infiltration and potentially diminishing chemotherapy efficacy (**Figure 1A**). Additionally, sensitivity to EGFR inhibitors was observed in the subset, offering valuable insights into potential clinical treatment strategies.

## 2 Materials and methods

### 2.1 Data acquisition and processing

snRNA-seq data was curated from GSE200256, also deposited in project SCPCP000006 (https://scpca.alexslemonade.org/projects/SCPCP000006) in the Single-cell Pediatric Cancer Atlas Portal (ScPCA) portal (25), encompassing 22 favorable and 18 anaplastic primary samples, 10 of which underwent relapse after treatment. ST data was also curated from project SCPCP000006 in the ScPCA portal, including 100 slices from 41 patients, among which 12 patients underwent further recurrence.

Additionally, bulk RNA sequencing data, mutation data and clinical profiles were obtained from Therapeutically Applicable Research to Generate Effective Treatments (TARGET)-WT dataset via R package TCGAbiolinks. Copy number data were retrieved from the TARGET-WT GDC data portal. Only samples collected at initial diagnosis in TARGET-WT dataset were kept. Furthermore, GSE31403 (26) and GSE10320 (27) cohorts were retrieved from GEO database, comprising 224 samples and 144 samples prior chemotherapy respectively.

### 2.2 Single-nuclei sequencing data processing and cell annotation

snRNA-seq data were analyzed using Seurat package version 5.0.1. Cells with less than 200 genes and genes expressed in less than 3 cells were removed. Following quality control, data normalization was performed using “NormalizeData” function utilizing default parameters. Variable genes were identified using the “FindVariableFeatures” function with the “vst” selection method and nfeatures=5000. Data were scaled using the “ScaleData” function, followed by principal component analysis (PCA) through “RunPCA”. Non-linear dimensionality reduction (uniform manifold approximation and projection, UMAP) was performed using the “RunUMAP” function. Batch effects were addressed with Harmony. Cell type annotations were carried out using canonical cell markers curated from previous research.

### 2.3 Identification of relapse-associated tumor subpopulations using Scissor algorithm

snRNA-seq data were integrated with bulk RNA-seq datasets and (TARGET-WT, GSE31403 and GSE10320) and phenotypic profiles using the “Scissor” package (28). Given the large number of cells, the pseudobulk method was initially employed to reduce the cell count. Briefly, we run the “FindClusters” with the resolution of 10. Cells from the same cluster and sample were subsequently merged into meta cells. This process reduced the number of cells from 161,635 to 4,274. The resulting meta cell matrix was constructed as a Seurat object and used as input for Scissor analysis. The alpha parameter was set to 0.05. Using TARGET-WT data with survival information, the Cox regression model was applied to identify Scissor+ cells associated with worse RFS. For the GSE31403 and GSE10320 bulk profiles, logistic regression models were applied to infer Scissor+ cells associated with relapse. The final Scissor+ cells, associated with the relapse phenotype and worse RFS, were defined as the intersection of Scissor+ cells identified across all three bulk datasets.

### 2.4 Functional enrichment analysis and developmental signatures of Scissor+ tumor cells

Scissor+ tumor signature genes were identified using Seurat’s “FindAllMarkers” and further filtered with the threshold |avg_log2FC|>=1 and p-value <0.05. Gene Ontology (GO) analysis and Kyoto Encyclopedia of Genes and Genomes (KEGG) analysis were conducted using R package “clusterProfiler”. Fetal ureteric bud (UB), cap mesenchyme (CM), and primitive vesicle (PV) gene sets were curated from previous published research (4). Subsequently, Seurat’s “AddModuleScore” function was employed to identify gene sets associated with fetal UB, CM, and PV in both Scissor+ and Scissor- tumor cells.

### 2.5 Spatial transcriptomics data processing

ST data were also curated from project SCPCP000006 in the ScPCA portal. 98 slides from 40 WT primary samples were kept. Raw matrices were processed using Seurat package version 5.0.1 for quality control, normalization, dimension reduction and Louvain clustering. Sample level normalization was performed using the SCTransform function in Seurat package. SpatialFeaturePlot function was used to visualize features.

### 2.6 Single sample gene set enrichment analysis

Single-sample gene set enrichment analysis (ssGSEA) was performed to identify distinct cell types within the spatial transcriptomics data. Specifically, tumor signatures for Scissor+ and Scissor- cells, along with cell type signatures derived from previous studies, were utilized. The ssGSEA algorithm from the GSVA package (version 1.38.2) was employed to calculate the expression levels of these curated signatures at each spatial location. The resulting data were further processed to assess correlations between different cell types and were visualized using spatial feature plots.

### 2.7 Analysis of cell–cell interactions

To analyze potential cell–cell interactions among distinct cell types in snRNA-seq dataset, the CellChat package (version 2.1.2) was employed to quantitatively infer and analyze intercellular communication networks (29). This algorithm utilized network analysis and pattern recognition approaches to predict the major incoming and outgoing signals for cells. Cell types with less than 100 cells were removed. Significant signaling pathways and ligand-receptor pairs were extracted based on permutation tests with a p<0.05.

### 2.8 Prediction of response and resistance to treatment

The PERCEPTION computational pipeline (https://github.com/ruppinlab/PERCEPTION) was employed to predict the response of tumor cells to chemotherapy and targeted therapies (30). PERCEPTION utilizes publicly available matched bulk and single-cell expression profiles derived from large-scale cell line drug screens, enabling the construction of treatment response models based on pseudobulk data in this study. A total of 44 drugs incorporated within the PERCEPTION models were analyzed. During the execution of the run_parallel_feature_ranking_bulk function, the parameter infunc_exclude_cancer was set to ‘PanCan’.

### 2.9 Ensemble model construction

An ensemble machine learning framework was applied to predict RFS using TARGET-WT data. First, the data were preprocessed by normalizing features, and relevant genes were selected through feature importance filtering. The ensemble was composed of four survival models, including Cox proportional hazards, random survival forests, support vector machines and XGBoost, using the R mlr3 and mlr3proba packages. For model training, 5-fold cross-validation was employed, and hyperparameter tuning was conducted using random search with 100 evaluations. Model performance was assessed via concordance index (C-index), Brier score, and survival calibration. Additionally, bootstrap sampling was used for robust validation and risk score prediction to ensure reliable feature selection and accurate stratification.

### 2.10 Immunohistochemistry

Consecutive 5-μm thick tissue sections were prepared from formalin-fixed, paraffin-embedded WT primary tumor tissues and processed for immunohistochemistry. Following deparaffinization, rehydration, and antigen retrieval, endogenous peroxidases and nonspecific binding were blocked. Sections were incubated overnight at 4°C with primary antibodies against TGFA (HUABIO, ET7107-40, diluted 1:400), followed by incubation with secondary antibodies. Nuclei were lightly counterstained with hematoxylin.

### 2.11 Statistical analyses

All statistical analyses were conducted using R software version 4.2.3. Kaplan-Meier survival curves were generated, and the log-rank test was employed. A p-value of <0.05 was deemed statistically significant. The chi-squared test was applied to compare categorical variables, and continuous variables were compared through the Wilcoxon test or t-test. Further statistical methods are detailed in the figure legends.

## 3 Results

### 3.1 Integration and exploration of WT single-cell atlas

To investigate transcriptional heterogeneity in WT at single-cell level, we utilized a published snRNA-seq dataset (SCPCP000006, ScPCA portal) which included 40 WT tumor samples, 39 of which were collected from the kidney at initial diagnosis, and one was collected from liver metastases at autopsy (**Figure 1A**). The data were processed using the Seurat pipeline. To minimize batch effects, the Harmony algorithm was employed for sample integration. A total of 199,441 cells were retained after quality control and visualized using UMAP plots, with color coding based on sample type, cell classification, histological subtype, sample collection time, or relapse status (**Figure 1B**, **Supplementary Figures S1A–D**) (4, 18, 31). Four primary cell populations were first identified using canonical cell markers: WT tumor cells (*WT1, NCAM1, SIX1, SIX2, PAX2*), cancer-associated fibroblasts (CAFs) (*ACTA2, TAGLN, COL1A1, PDGFRB*), endothelial cells (*PECAM1, CLDN5, ENG, VWF*) and immune cells. Subsequently, immune cells were further subdivided and reanalyzed to enable a more comprehensive annotation. Eight immune subsets were identified, including T cells (*CD3E, CD3D, CD8A*), NK cells (*GZMK, NKG7, GZMA, KLRD1*), B cells (*CD79A, CD79B, MS4A1, IGHM*), monocyte-macrophages (*CD68, CD14, LYZ, C1QC, CD163*), neutrophils (*S100A8, S100A9, CSF3R*), canonical dendritic cells (cDC) (*CD1C, FCER1A, CLEC9A, HLA-DQA1*), plasmacytoid dendritic cells (pDC) (*LILRA4, IL3RA, GZMB*), and mast cells (*KIT, TPSAB1, CPA3*) (**Figures 1B–D**). Copy number variation (CNV) inferred from the snRNA-seq data revealed that some tumor cells exhibited deletions on chromosomes 11,14 and 16, while non-tumor cells showed no apparent CNVs (**Supplementary Figure S2**). Due to the low recovery of immune cells in snRNA-seq platform (32), tumor cells and stromal cells were more prevalent than immune cells. Tumor cells were found to be more abundant in samples with anaplastic histology and in patients who experienced recurrence following initial treatment, whereas stromal and immune cells demonstrated the opposite trend (**Figures 1E, F**).

### 3.2 Unraveling the heterogeneity of Wilms tumor cells

**Figure 2:**
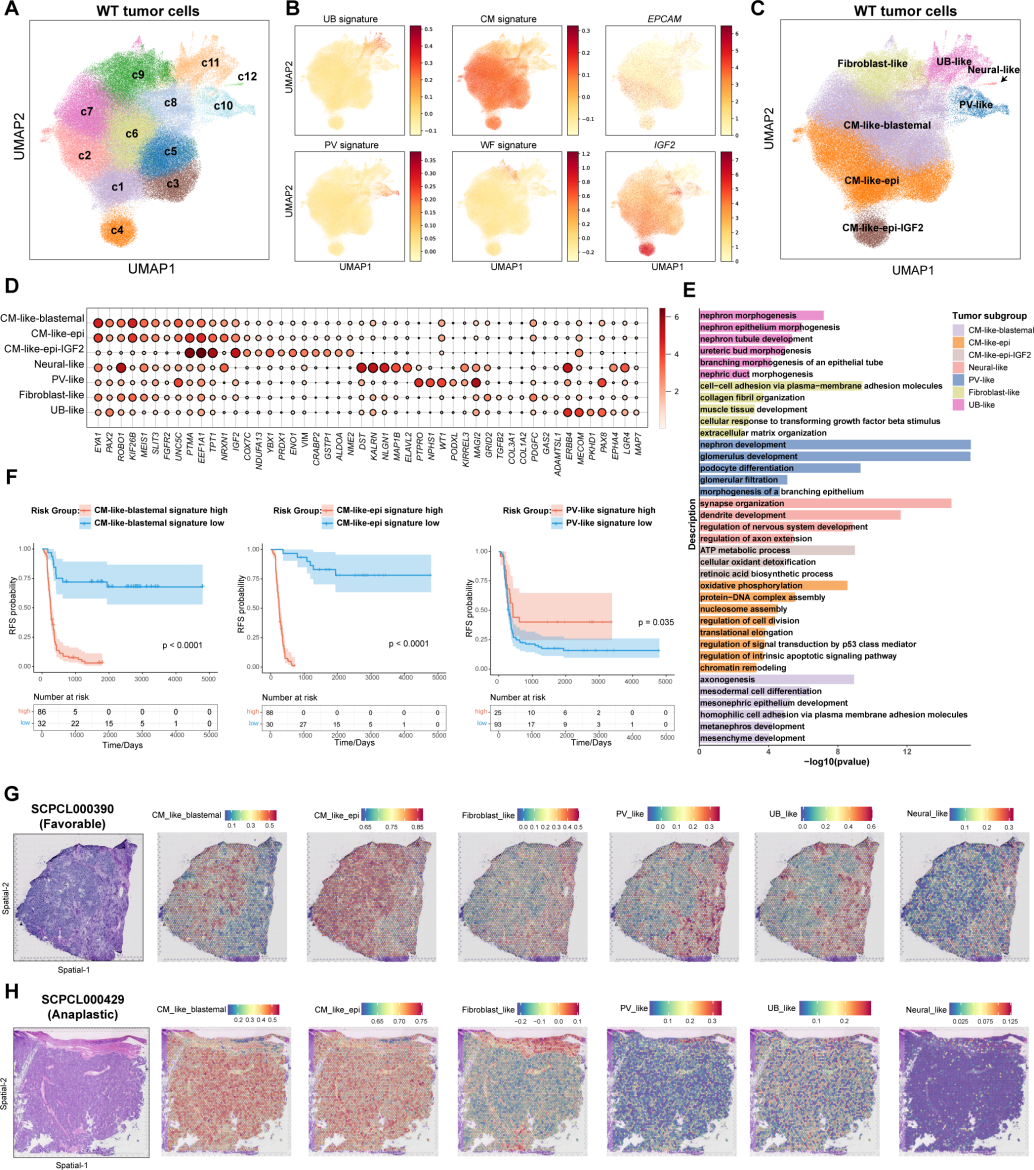
Identification of distinct tumor subgroups in WT. **(A)** The UMAP visualization of twelve tumor clusters derived from unsupervised clustering analysis. **(B)** The UMAP visualization of fetal kidney signatures (UB, ureteric bud; CM, cap mesenchyme; PV, primitive renal vesicles), Wilms tumor fibroblast signature, and selected gene expression. **(C)** The UMAP visualization of seven tumor subgroups. **(D)** The dot plot of selected DEGs across all tumor subgroups. **(E)** The bar plot displaying the GO gene enrichment results for top 100 DEGs in each tumor subgroup. **(F)** Kaplan-Meier survival curves showing relapse-free survival in the TARGET-WT cohort, stratified by high and low expression of selected tumor subgroup signatures (log-rank test). **(G)** Hematoxylin and eosin (H&E) staining of sample SCPCL000390 (favorable histology) (Left). Spatial distribution of tumor subgroup signatures quantified by ssGSEA(Right). **(H)** H&E staining of sample SCPCL000429 (unfavorable histology) (Left). Spatial distribution of tumor subgroup signatures quantified by ssGSEA(Right).

To characterize the transcriptional heterogeneity within WT tumor cells, we conducted clustering analysis, identifying twelve distinct cell clusters (c1–c12) (**Figure 2A**). The normal nephrogenesis is initiated by ureteric bud (UB), around which nephron progenitor cells (NPCs) condense to form a cap mesenchyme (CM) around the UB. Subsequently, these NPCs give rise to primitive renal vesicles (PV) (33). Previous studies have demonstrated that WT tumor cells mimic fetal kidney cell types, such as UB, CM, and PV cells, with some tumor cells also exhibiting characteristics resembling stromal fibroblast-like cells (4). Thus, we measured published fetal UB, CM, PV and fibroblasts-like WT signatures in all tumor cells (**Supplementary Table S1**). Interestingly, the expression of these four signatures was largely mutually exclusive, allowing us to group the twelve clusters into four major categories: Fibroblast-like, CM-like, UB-like, and PV-like cells (**Figures 2B, C**). Additionally, cluster c12, which expressed neural-related genes (e.g., *DST, MAP2, ELAVL2*), was classified as neural-like cells (**Figures 2C, D**).

WT histology typically exhibits a triphasic pattern, comprising epithelial, stromal, and blastemal components (1). To further analyze these components, we examined the expression of canonical markers used in histological classification of WT, including *EPCAM, KRT18, KRT8, CDH6, MME, TJP1* for epithelial components, *NCAM1, SIX1, SIX2, PAX2, EYA1, SALL1* for blastemal components, and *COL1A1, COL1A2, COL3A1* for stromal components (**Supplementary Figures S3A, B**). While stromal components were clearly characterized by fibroblast-like tumor cells, canonical epithelial and blastemal markers were predominantly mixed within CM-like cells. Epithelial markers such as *CDH6* and *MME* were mainly expressed in PV-like cells (**Supplementary Figures S3A, B**). Tumor clusters c1–c4 exhibited relatively high expression of *EPCAM* and *KRT18*, with c4 showing elevated *IGF2* expression (**Figure 2B**). Tumor clusters c5-c8 expressed higher levels of blastemal markers, such as *EYA1* and *PAX2* (**Figure 2D**). Consequently, we refined subgroup definitions: c1-c3 as CM-like-epithelial (CM_like_epi), c4 as CM-like-epithelial with IGF2 high expression (CM_like_epi_IGF2), and c5-c8 as CM-like-blastemal (CM_like_blastemal) (**Figure 2C**). In total, seven subgroups were identified. UB_like and fibroblast-like cells were WT weak positive (**Supplementary Figure S3C**). CM_like_epi cells exhibited relatively higher necrosis signature (**Supplementary Figure S3D**).

We conducted differential expressed gene (DEG) analysis for each tumor subgroup and performed Gene Ontology (GO) enrichment analysis (**Supplementary Table S2**, **Figure 2E**). The results largely validated the identity and functional characteristics of each subgroup. For instance, fibroblast_like cells were enriched for genes associated with the extracellular matrix, while neural_like cells exhibited neuronal-related features. CM_like_blastemal cells enriched for mesonephric and metanephric developmental markers, indicating their role as progenitor cells in early kidney development. CM_like_epi cells showed signatures related to ATP metabolism, oxidative phosphorylation, and cell proliferation. In contrast, PV_like cells were enriched for genes related to nephron development and podocyte differentiation. Although UB_like cells exhibited higher UB signatures, they also up-regulated genes involved in nephric duct morphogenesis and epithelial tube branching process. These suggested PV_like and UB_like cells represent more mature epithelial cells (**Figure 2E**).

We further examined the distribution of tumor subgroups across different samples and clinical categories (**Supplementary Figures S3E, F**). Notably, CM_like_epi_IGF2 was primarily derived from sample SCP000182, whereas other subgroups were present across multiple samples. CM_like_blastemal and fibroblast-like cells were enriched in samples that later experienced recurrence, while UB-like and PV-like cells were more abundant in samples that did not recur (**Supplementary Figure S3E**). Consistent with these distributions, Kaplan-Meier survival analysis demonstrated that patients with higher levels of CM_like_blastemal, CM_like_epi, or fibroblast-like features at diagnosis had significantly worse RFS, whereas patients with higher PV_like features exhibited significantly better RFS (**Figure 2F**, **Supplementary Figure S3G**).

Finally, we sought out to visualize these tumor subgroups in ST data. To achieve this, we analyzed published spatial transcriptomes from 98 slides of 40 WT primary samples using 10X Visium platform (Project SCPCP000006 in the ScPCA portal). CM_like_blastemal cells were localized in the blastemal region of the tissue, while fibroblast-like cells overlapped with mesenchymal stroma (**Figures 2G, H**). PV-like and UB-like cells were co-localized with epithelial tubular and glomerular structures in the stroma. CM_like_epi cells were observed in both the blastemal and epithelial regions, suggesting a mixed phenotype. Neural-like cells, being relatively sparse, were dispersed throughout the tissue sections (**Figures 2G, H**, **Supplementary Figures S3H, I**).

### 3.3 Identification of Scissor+ tumor cells linked to WT recurrence

Given the critical role of tumor cell phenotypes in WT recurrence, and the recognition that recurrence is not restricted to specific histological subtypes, we performed Scissor analysis (28), a method designed to objectively and systematically identify the tumor cells most strongly associated with WT relapse from snRNA-sea data. This analysis integrated snRNA-seq datasets with the TARGET-WT dataset, which contains RFS information (n=118), along with two additional bulk RNA sequencing cohorts, GSE31403 (n=224) (26) and GSE10320 (n=144) (27), which provide clinical information on relapse status.

**Figure 3:**
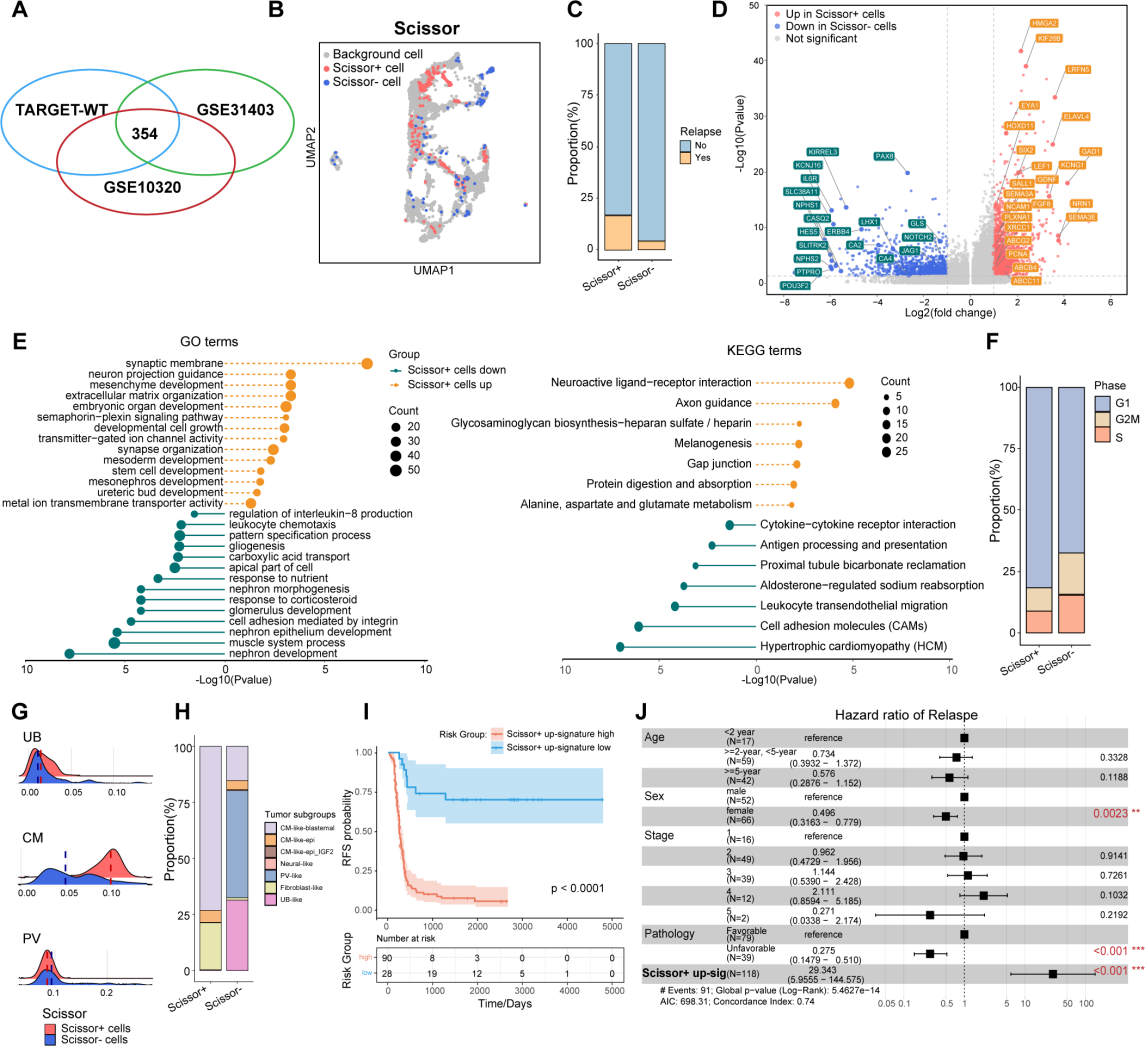
Identification of Scissor+ tumor cells associated with poor relapse-free survival. **(A)** Venn diagram showing 354 common cells identified through Scissor analysis, shared among the TARGET-WT, GSE31403, and GSE10320 datasets. **(B)** UMAP visualization of tumor meta cells from Scissor analysis, with Scissor+ cells in red and Scissor- cells in blue. **(C)** The volcano plot showing the differentially expressed genes between Scissor+ and Scissor- tumor cells. Genes with |avg_logFC|>=1 and P <0.05 were highlighted. **(D)** The bar plot depicting the proportions of tumor cells from samples with or without further recurrence in Scissor+ or Scissor- tumor cells. **(E)** The lollipop plot showing enriched GO and KEGG pathways in Scissor+ and Scissor- tumor cells. **(F)** The bar plot comparing the distributi on of cell cycle phases between the Scissor+ and Scissor- tumor cells. **(G)** The ridge plot illustrating fetal kidney cell signatures, including ureteric bud (UB), cap mesenchyme (CM), and primitive vesicles (PV). **(H)** The bar plot comparing the distribution of tumor subgroups between the Scissor+ and Scissor- tumor cells. **(I)** Kaplan-Meier survival curves showing relapse-free survival in the TARGET-WT cohort, stratified by high and low expression of Scissor+ tumor up-regulated genes (log-rank test). **(J)** Multivariable Cox regression analysis of RFS in the TARGET-WT cohort, presented as hazard ratios with 95% confidence intervals.

Initially, we conducted pseudobulk aggregation analysis on all tumor cells to generate meta cells, thereby reducing the total cell count and enhancing compatibility between snRNA-seq and bulk RNA datasets. A total of 161,635 tumor cells were merged into 4,247 meta cells for subsequent Scissor analysis (**Supplementary Figures S4A, B**). Scissor+ cells were classified as positive, while Scissor- cells were defined as negative across all three bulk RNA datasets (**Figure 3A**). This analysis identified 231 Scissor+ meta cells associated with relapse and poor RFS, whereas 123 Scissor- cells were linked to favorable RFS (**Figure 3B**). Consistently, the Scissor+ group contained a significantly higher number of cells from samples that later experienced relapse (*p*<2.2e-16, Chi-squared test) (**Figure 3C**). DEG analysis indicated that compared to Scissor- tumor cells, Scissor+ tumor cells exhibited significantly higher expression of genes involved in metanephros (*GDNF, KIF26B, EYA1, FGF2, HOXD11*) and mesenchyme development (*SIX2, HMGA2, PDGFC, WNT3A, GATA4*), as well as semaphorin-plexin pathways (*SEMA3D, SEMA3A, SEMA3E, PLXNA1*), which are essential for regulating early fetal kidney morphogenesis (**Figures 3D, E**). Notably, Scissor+ also significantly upregulated CSCs markers previously revealed in WT, such as *SIX2, NCAM1, PROM1 (* 16–18), as well as ATP-binding cassette (ABC) transporters genes (*ABCG2, ABCB4, ABCC11*) and DNA repair pathways genes (*XRCC1, PCNA*), which associated with increased drug efflux activity and drug resistance (34, 35) (**Figures 3D, E**, **Supplementary Table S3**). Scissor+ tumor cells contained significantly fewer G2M/S phase cells and more G1 phase cells (*p*=0.0097, Chi-squared test), consistent with the relatively quiescent nature of CSCs (**Figure 3F**). Conversely, downregulated genes were linked to glomerulus development (*PAX8, PTPRO, KIRREL3, LHX1, NPHS1, NOTCH2, JAG1*), muscle system process (*NPNT, ACTA1*), and proximal tubule bicarbonate reclamation pathways (*GLS, CA2, CA4*) (**Figures 3D, E**, **Supplementary Table S3**).

We further compared the distribution of fetal kidney signatures and tumor subgroups between Scissor+ and Scissor- cells (**Figures 3G, H**). The analysis revealed that the cells identified by Scissor, through an unbiased and automated approach, were closely linked to the tumor subgroups manually defined. Consistent with their gene expression patterns, Scissor+ tumor cells, exhibiting enhanced CM signatures, were predominantly derived from CM_like_blastemal and fibroblast_like subgroups. In contrast, Scissor- tumor cells, showing increased PV features, contained more cells from PV_like and UB_like subgroups (*p*<2.2e-16, Chi-squared test) (**Figure 3H**). Collectively, Scissor+ cells may represent a group of CSCs with NPC-like characteristics, while Scissor- cells may correspond to more differentiated nephron epithelial cells.

We next curated the Scissor+ signature from up-regulated genes of Scissor+ tumor cells. Consistently, Kaplan-Meier analysis showed that patients with a higher Scissor+ signature had significantly worse RFS (**Figure 3I**). Moreover, in multivariate Cox regression analysis, the Scissor+ signature was identified as a statistically significant prognostic feature independent of age, sex, stage, and histology (**Figure 3J**).

In summary, Scissor+ tumor cells, which exhibited features of CSCs and retained transcriptional features of NPCs, were identified as being associated with WT relapse and poor RFS.

### 3.4 Establishment of predictive machine-learning model for WT relapse-free survival

**Figure 4:**
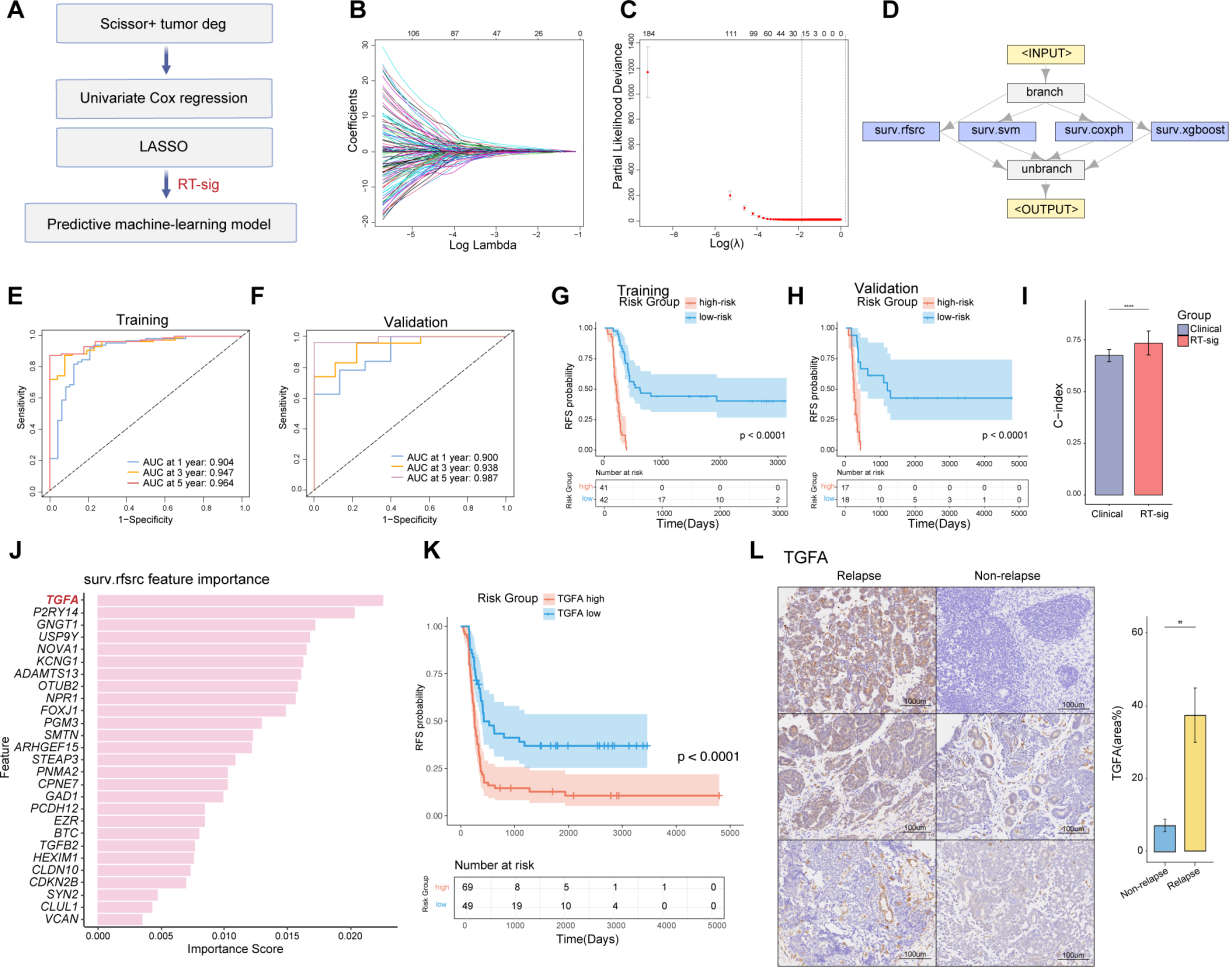
Construction of an ensemble machine-learning model for predicting WT RFS. **(A)** Schematic of the machine-learning framework. **(B)** Lasso coefficients for prognostic genes in the TARGET-WT cohort, with lines of different colors representing individual genes. **(C)** Determination of the optimal λ, selected when the partial likelihood deviance reached its minimum value in ten-fold cross-validation (data presented as mean with 95% confidence intervals). **(D)** Framework of the ensemble machine-learning model. **(E)** Time-dependent receiver operating characteristic (ROC) curves for predicting RFS at 1, 3, and 5 years in the training dataset. **(F)** Time-dependent ROC curves for predicting RFS at 1, 3, and 5 years in the validation dataset. **(G)** Kaplan-Meier survival curves illustrating relapse-free survival in the training dataset, stratified by high- and low-risk groups defined by the ensemble model (log-rank test). **(H)** Kaplan-Meier survival curves illustrating relapse-free survival in the validation dataset, stratified by high- and low-risk groups defined by the ensemble model (log-rank test). **(I)** Bar plot comparing the performance of the RT-sig model to combined clinical variables (sex, age, stage, histology) for predicting prognosis in the TARGET-WT cohort (two-sided Wilcoxon test, **** indicates p<0.0001). **(J)** Histogram displaying the feature importance from random forest survival model used in ensemble model construction. **(K)** Kaplan-Meier survival curves illustrating relapse-free survival in the TARGET-WT cohort, stratified by high- and low-expression of TGFA (log-rank test). **(L)** Immunohistochemistry images and bar plot illustrating TGFA expression in WT samples with or without relapse after initial treatment under the SIOP protocol. WT samples were collected from Children’s Hospital of Fudan University (two-sided Wilcoxon test, ** indicates p < 0.01).

To further translate features of Scissor+ tumor cells into diagnostic clinical applications, we developed a predictive machine-learning model to estimate patient RFS. The model was constructed using a multi-step feature selection and ensemble pipeline (**Figure 4A**). Univariate Cox regression analysis was first conducted to identify prognosis-related genes from the DEGs in Scissor+ cells, with a p-value threshold set at < 0.05. Mitochondrial and ribosomal genes were excluded, resulting in the selection of 2,537 genes. Following the removal of highly correlated genes, the Least Absolute Shrinkage and Selection Operator (LASSO) analysis was further applied to reduce the number of genes (**Figures 4B, C**). Ultimately, 27 genes were selected and constituted as a relapse-associated signature (RT-sig) for establishing the predictive ensemble machine-learning model using the mlr3 framework. Of these, 16 genes were classified as risk factors (HR>1.0), and 11 as protective factors (HR<1.0) (**Supplementary Table S4**). The ensemble model consisted of four algorithms acting in operating including random survival forest, support vector machine, Cox proportional hazards model and XGBoost (**Figure 4D**). The TARGET-WT (n=118) cohort was divided into a training set (n = 83) and a validation set (n = 35) at a ratio of 7:3. Additionally, 5-fold cross-validation was conducted to optimize hyperparameters for each algorithm. Time-dependent receiver operating characteristic curve (ROC) analysis revealed the 1-, 3-, and 5-year areas under the curves (AUC) were 0.904, 0.947, and 0.964 in the training set (**Figure 4E**) and 0.900, 0.938 and 0.987 in the validation set (**Figure 4F**). Patients were classified into high- and low-risk groups based on median crank value derived from the ensemble model. Kaplan-Meier analysis demonstrated that high-risk patients had significantly worse RFS than low-risk patients (**Figures 4G, H**).

We further compared the predictive performance of RT-sig with clinical characteristics including gender, stage, histology, and age. A bootstrap test was employed. The results showed that the C-index of RT-sig was significantly higher than that of the combined clinical characteristics, indicating it exhibited superior accuracy in predicting WT RFS (**Figure 4I**). A comprehensive assessment of the importance of RT-sig genes within the random forest survival algorithms was conducted during the ensemble model training process. This revealed that *TGFA* emerged as the most significant gene associated with patient outcomes and high expression of *TGFA* indicated worse RFS (**Figures 4J, K**). Given its prominence, TGFA was selected for subsequent immunohistochemistry experiments. Notably, the expression levels of TGFA were markedly elevated in primary tumors of WT patients who subsequently experienced disease recurrence (**Figure 4L**). This result suggested the potential of TGFA as a biomarker for predicting poor RFS in clinical settings.

### 3.5 Genomic alterations underlying RT-sig-based risk stratification

**Figure 5:**
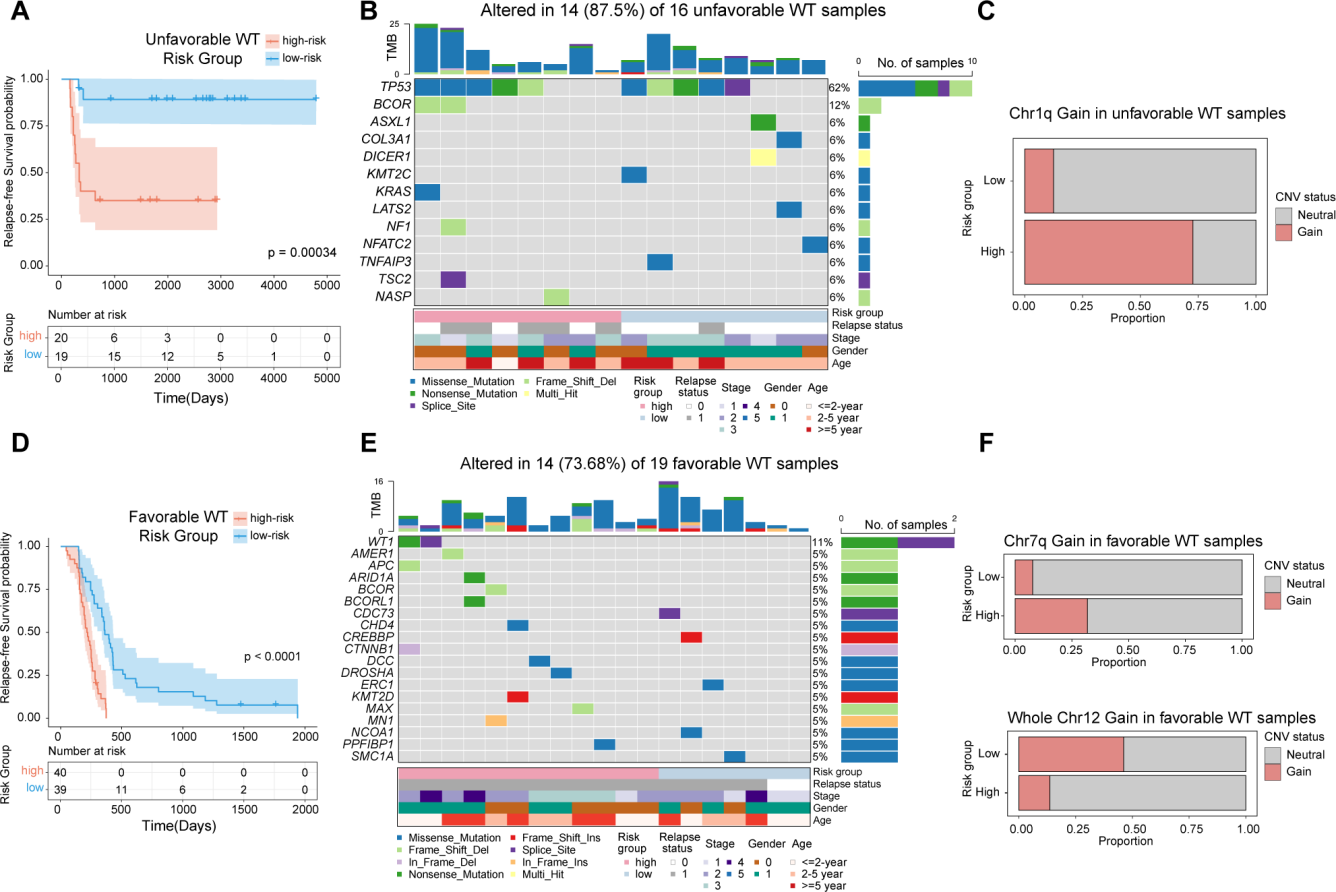
Genomic alterations underlying RT-sig-based risk stratification in unfavorable and favorable WT. **(A)** Kaplan-Meier survival curves illustrating RFS in unfavorable WTs (n=39), stratified into high- and low-risk groups as defined by the ensemble machine learning model (log-rank test). **(B)** Oncoplot showing the mutations in a selection of frequently mutated genes in unfavorable WTs with publicly available paired RNA-seq and mutation data (n=16). **(C)** Barplot depicting the proportion of unfavorable WT samples with publicly available paired RNA-seq and copy number data showing chromosome 1q gain in high-risk (8/11) and low-risk (1/8) groups. **(D)** Kaplan-Meier survival curves illustrating RFS in favorable WTs(n=79), stratified into high- and low-risk groups as defined by the ensemble machine learning model (log-rank test). **(E)** Oncoplot showing the mutations in a selection of frequently mutated genes in favorable WTs with publicly available paired RNA-seq and mutation data (n=19). **(F)** Barplot depicting the proportion of favorable WT samples with publicly available paired RNA-seq and copy number data showing chromosome 7q gain and whole chromosome 12 gain in high-risk (7q gain:7/22; chr12 gain:3/22) and low-risk groups (7q gain:2/26; chr12 gain:12/26).

We further explored genomic alterations potentially associated with RT-sig-based risk stratification. Given that previous studies have shown distinct genomic alterations in favorable and anaplastic tumors (31, 36), we employed the ensemble machine learning model to stratify the risk groups within anaplastic and favorable histology tumors separately (**Figure 5A**). TARGET-WT patients with paired, publicly available transcriptomic, mutation, or copy number data were analyzed.

*TP53* mutations are known to be associated with anaplasia in WT, which confers a high relapse risk and poor prognosis (31). In our analysis of unfavorable WT cases, we observed that both high- and low-risk subgroups carried *TP53* mutations, highlighting its pivotal role in the development of unfavorable WTs (**Figure 4B**). Notably, within the high-risk subgroup of unfavorable WTs, recurrent mutations in *BCOR* were identified, further emphasizing the complex genetic landscape of these tumors (**Figure 5B**). In addition, this subgroup exhibited a significantly higher proportion of samples with segmental gains of chromosome 1q (*p*=0.020, two-sided Fisher’s exact test) (**Figure 5C**). The gain of 1q is a well-established adverse prognostic marker in WT, contributing to its aggressive clinical course (37).

In favorable WTs, although no TP53 mutations were observed in either subgroup, high-risk tumors harbored more mutations in previously identified WT-related genes, such as *WT1*, *AMER1*, *BCOR*, *CDC73*, *ARID1A*, *CTNNB1* and *DROSHA* (36), all of which have been implicated in the development and progression of WT (**Figures 5D, E**). Furthermore, segmental gains of chromosome 7q were predominantly found in the high-risk group (*p*=0.038, two-sided Fisher’s exact test), whereas whole chromosome 12 gain was more frequently observed in the low-risk group (*p*=0.027, two-sided Fisher’s exact test) (**Figure 5F**). These findings are consistent with recent studies suggesting that chromosome 12 gain serves as a marker of favorable prognosis in WT patients (38).

In summary, our results underscore the utility of the ensemble machine learning model for RFS based on RT-sig, which not only predicts clinical outcomes but also reflects underlying genomic alterations, providing insights into both adverse and favorable genetic profiles in WT patients.

### 3.6 Analysis of distinct intercellular communication between Scissor+ and Scissor- tumor cells

**Figure 6:**
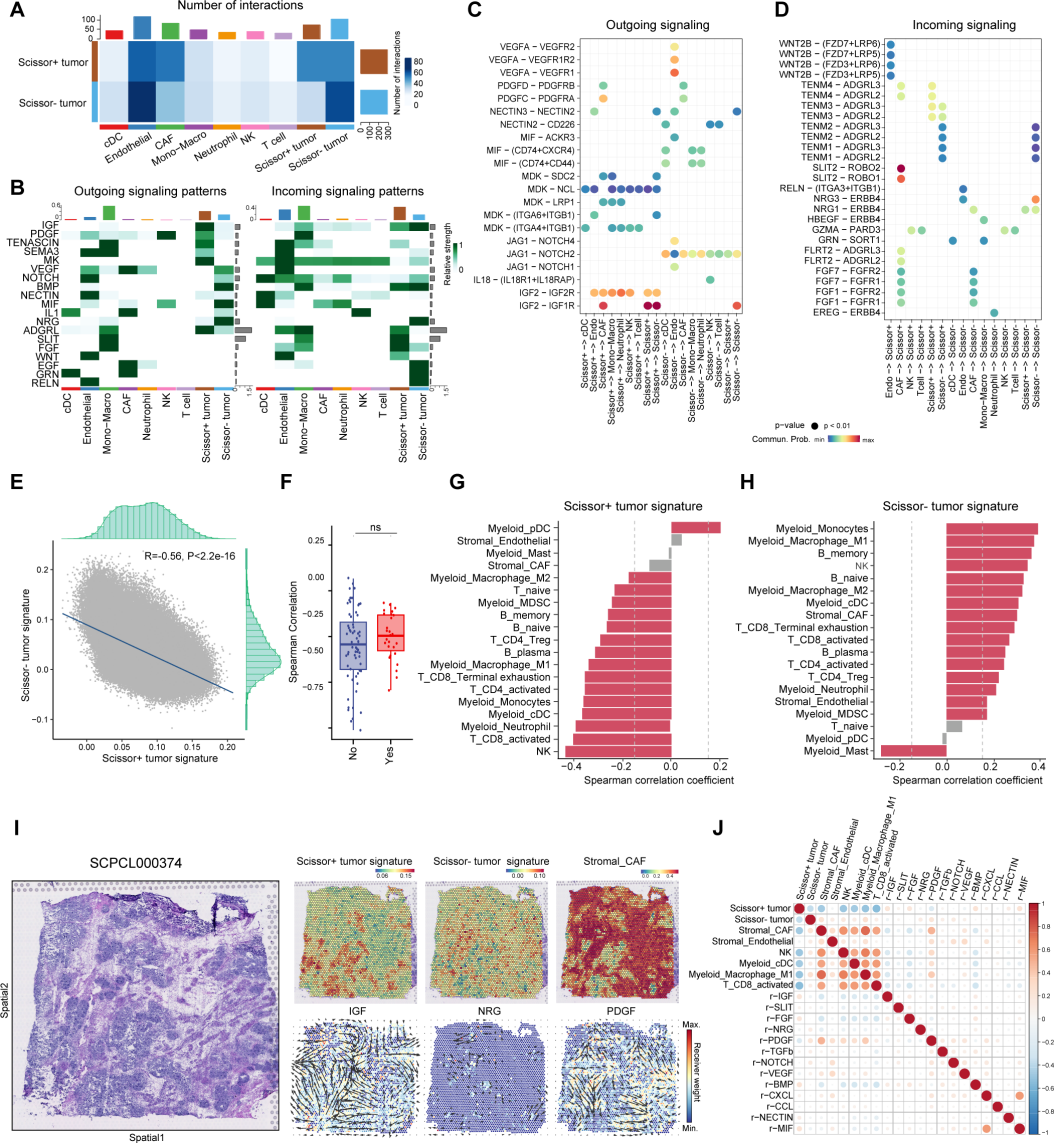
Identification of specific intercellular communications between Scissor+ tumor cells and CAFs in WT. **(A)** Heatmap showing the overall number and strength of intercellular communications in Scissor+ and Scissor tumor cells. **(B)** Heatmap showing the major outgoing and incoming signaling pathways. **(C)** Dot plot illustrating significant ligand-receptor pairs from outgoing signaling pathways revealed in **(B)**. **(D)** Dot plot illustrating significant ligand-receptor pairs from incoming signaling pathways revealed in **(B)**. **(E)** Scatter plot showing the negative correlation between Scissor+ and Scissor- tumor signature in spatial transcriptomics. **(F)** Box plot showing the spearman correlation coefficients between Scissor+ and Scissor- tumor signature in spatial transcriptomics from samples with or without further recurrence. (Two-sided Wilcoxon test). **(G)** Bar plot displaying the spearman correlation between Scissor+ tumor signature and features of other cell types (|Correlation coefficient|>0.2 and p value <0.05 was highlighted in red). **(H)** Bar plot displaying the spearman correlation between Scissor- tumor signature and features of other cell types (|Correlation coefficient|>0.2 and p value <0.05 was highlighted in red). **(I)** Hematoxylin and eosin (H&E) staining of sample SCPCL000374 (favorable histology with further relapse) (Left). Spatial distribution of cell type signatures (Scissor+ tumor, Scissor- tumor and CAF signatures) and inferred signaling pathways in ST from commot analysis colored by receiver weight (Right). Arrows indicate the spatial direction of the pathways. **(J)** Spearman correlation coefficients for cell type signatures and spatial receiver weights of signaling pathways.

After analyzing the signatures of Scissor+ tumor cells, we next investigated whether the interaction between tumor cells and tumor microenvironment (TME) cells plays a critical role in WT chemotherapy resistance. To address this, we performed Cellchat analysis (29) to infer and analyze intercellular communication networks underlying distinct tumor phenotypes. Compared to Scissor- cells, Scissor+ tumor cells exhibited more interactions with CAFs, while demonstrating less communication with endothelial cells and immune cells, including cDCs, NK cells, and T cells (**Figure 6A**). We further examined the relative signaling strength of various pathways across all cell types (**Figure 6B**). For outgoing signals, pathways that induce CAFs, including IGF2 and PDGF pathways, were primarily received by fibroblasts from Scissor+ cells (39, 40). The ligand-receptor pairs IGF2-IGF1R, PDGFD-PDGFRB and PDGFC-PDGFRA were enriched between these two cell types, implicating Scissor+ tumor cells may play an important role in promoting expansion of CAFs (**Figure 6C**). Conversely, Scissor- tumor cells displayed a pro-angiogenic and immune-activating phenotype by eliciting NOTCH/VEGF signaling and NECTIN/MIF/IL1 signaling, respectively. Ligand-receptor pairs JAG1-NOTCH2/4, VEGFA-VEGFR1, NECTIN2-CD226, MIF-CD74+CXCR4/CD44, IL18-(IL18R1+IL18RAP) were enriched correspondingly (**Figure 6C**). Regarding incoming signaling, Scissor+ cells mainly received signals from CAFs, including SLIT, FGF, and ADGRL pathways (**Figure 6B, D**). The SLIT2-ROBO1/2 interaction has been shown to driver tumor immunosuppression and progression (41). The activation of FGF/FGFR signaling is crucial not only for nephron progenitor cell development (39, 42), but also for WT tumorigenesis (43). More importantly, IGF2 secreted by Scissor+ tumor cells could act on themselves through IGF2-IGF1R/IGF2R interactions (**Figure 6D**). Loss of imprinting of the IGF2 gene is the most common epigenetic alteration in WT and activation of IGF2 pathway has been associated with drug resistance in several tumors (44). Meanwhile, Scissor- tumor cells mainly received signals through Neuregulin-ERBB4 interactions with endothelial cells, and GRN-SORT1 interactions between cDCs/mono-macrophages (**Figure 6D**).

Since Scissor+ and Scissor- cells exhibited different interactions with TME cells, we then asked whether they would be spatially organized within WT. By leveraging single sample gene set enrichment analysis (ssGSEA), we scored Scissor+ and Scissor- cell signatures alongside the curated immune and stromal gene sets in ST data to identify spatial distribution of each cell type (**Supplementary Table S5**, **Supplementary Figure S5**) (31, 45–47). Notably, the Scissor+ and Scissor- signatures exhibited strong negative correlations, irrespective of relapse status, suggesting that these distinct cancer phenotypes occur in mutually exclusive regions of WT. (**Figures 6E, F**).

We further calculated spatial correlation between Scissor+/Scissor- tumor cells and non-malignant cell types across all spots. Scissor+ tumor cells exhibited negative correlation with nearly all immune cell types, whereas Scissor- tumor cells showed the opposite trend (**Figures 6G, H**). Compared to samples without relapse, in slides from samples which further underwent recurrence, Scissor+ tumor cells were more closely clustered and resided in immune-desert niches surrounded by fibroblast septa, potentially hindering immune cell infiltration (**Supplementary Figure S6**). In contrast, Scissor- cells were more diversely distributed, resided in immune-enriched areas with abundant T cells, myeloid and endothelial cells (**Figure 6I**, **Supplementary Figures S5, S6**). We also dissected cellular interactions in ST and determine the spatial distribution of signaling pathways. Consistent with snRNA results, ST analysis inferred higher receiver signals for the SLIT, IGF and FGF pathways in Scissor+ enriched areas, while NRG pathway receiver signal was abundant in Scissor- enriched regions (**Figures 6I, J**, **Supplementary Figure S5**). Notably, CAF-inducing pathways (e.g., PDGF, TGFb) were enriched at the interface between Scissor+ tumor cells and CAFs, highlighting these signals may serve as key mediators in the dynamic crosstalk between Scissor+ tumor cells and surrounding stromal components (**Figures 6I, J**).

### 3.7 Inference of response and resistance to treatment

**Figure 7:**
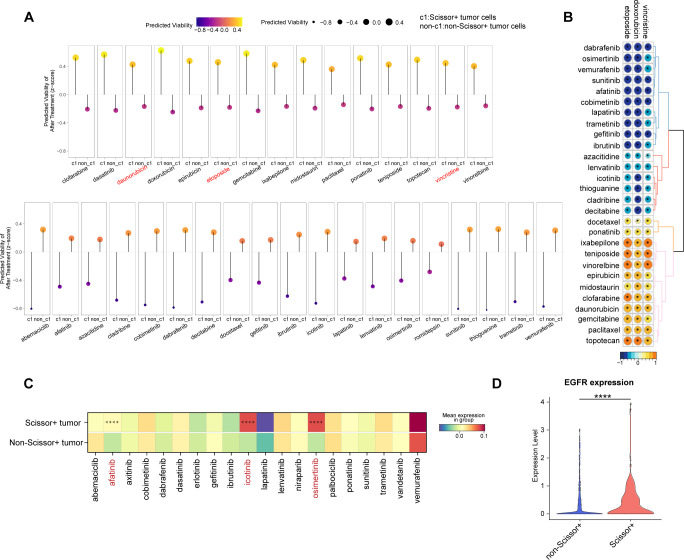
Prediction of drug response in WT patients. **(A)** Predicted cell viability after treatment in Scissor+ and non-Scissor+ tumor cells. The upper row shows drugs that are more effective in non-Scissor+ tumor cells, while the lower row displays drugs that are more effective in Scissor+ tumor cells. **(B)** Correlation matrix showing associations among drugs used in WT regimens (vincristine, doxorubicin, etoposide) and other drugs in the PERCEPTION database (* indicates p < 0.05). **(C)** Heatmap of drug target expression levels in Scissor+ and non-Scissor+ tumor cells (**** indicates p < 0.0001, two-sided Wilcoxon test). **(D)** Violin plot comparing EGFR expression in Scissor+ and non-Scissor+ tumor cells (**** indicates p < 0.0001, two-sided Wilcoxon test).

Finally, to investigate potential drug sensitivities in Scissor+ tumor cells, we conducted the PERCEPTION analysis based on publicly available expression data from large-scale cell-line drug screens (30). For each sample, we predicted the treatment response for Scissor+ and Scissor- tumor cells separately. This analysis revealed that, compared to Scissor- tumor cells, Scissor+ tumor cells are predicted to be less responsive to all chemotherapy agents, including vincristine, doxorubicin, and etoposide, which are part of the COG and SIOP regimens (**Figure 7A**). This finding further supports the results that Scissor+ tumor cells are resistant to chemotherapy and may contribute to WT recurrence. Notably, Scissor+ tumor cells demonstrated responsiveness to targeted therapies (**Figure 7A**). We also calculated PERCEPTION predictions for each drug and the correlations of drug sensitivity predictions between WT associated drugs (vincristine, doxorubicin, and etoposide) and other therapeutic agents (**Figure 7B**). These results suggest that resistance to WT-associated drugs may confer cross-sensitivity to targeted therapies.

We further examined the expression of potential targets for each targeted drug. The average expression levels of target genes for afatinib, icotinib, and osimertinib were significantly higher in Scissor+ tumor cells (**Figure 7C**, **Supplementary Table S6**). Notably, the target *EGFR* was common among the three drugs, with significantly higher expression observed in the Scissor+ group (**Figure 7D**). These findings suggest that patients at higher risk of relapse may benefit from a combined therapeutic approach, incorporating both conventional chemotherapy and EGFR-targeted therapies.

## 4 Discussion

WT is the most common renal tumor in childhood, with tumor recurrence associated with increased mortality and remained as a significant concern. However, the tumor phenotypes contributing to recurrence and the predictors of relapse at diagnosis have not been fully elucidated. In this study, we first characterized the transcriptional heterogeneity of WT tumor cells, classifying them into CM-like-blastemal, CM-like-epi, fibroblast-like, PV-like, UB-like, and neural-like cell populations. Utilizing the unbiased automatic Scissor algorithm, we discovered a subset of Scissor+ tumor cells associated with poor RFS, primarily originating from CM-like-blastemal and fibroblast-like cells. Specific cellular interactions and spatial distributions of recurrence-associated tumor cells were characterized. The dynamic crosstalk between these tumor cells and CAFs may play a critical role in immunosuppression and facilitate tumor persistence. Furthermore, an ensemble machine learning model was constructed to predict WT RFS at diagnosis, which could be used in diagnostic clinical applications. Conventional chemotherapy and EGFR-targeted therapies aimed at Scissor+ tumor cells could potentially overcome resistance mechanisms and prolong RFS in WT patients.

WT has been revealed to be originate from aberrant fetal kidney cells (4). Currently, the COG defines WT pathology using two histological classifications: favorable histology and unfavorable histology, the latter indicating the presence of anaplasia. Classical histological features of WT include a triphasic pattern comprising epithelial, stromal, and blastemal components (48). Immunohistochemistry, employing specific markers for each component, is commonly used for histological classification and risk stratification. However, the expression of epithelial and blastemal markers in snRNA-seq data is intermixed within cell populations. In our study, we found that, rather than relying on traditional immunohistology markers, signatures derived from fetal kidney cell types were more effective in categorizing tumor cells, and the resulting cell subgroups correlated with patient prognosis. Diagnostic markers derived from fetal kidney data and tumor subgroups may potentially enhance both the diagnosis and precise classification of WT.

CSCs have been revealed to be responsible for therapy resistance and tumor recurrence, which are capable of persisting after chemotherapies, and maintaining self-renewal and differentiating into the heterogeneous nontumorigenic cancer cells that comprise most of the tumor bulk (49, 50). Previous studies have identified SIX2+CITED1+, PROM1+ or NCAM1+ALDH1+ tumor cells as potential CSC-like populations in WT (16–18). In this study, relapse-associated Scissor+ tumor cells were consistent with previous studies, showing significant upregulation of CSC markers SIX2, PROM1, and NCAM1. Notably, Scissor+ tumor cells, primarily derived from CM-like-blastemal and Fibroblast-like tumor subgroups, exhibited characteristics of CM nephron progenitor cells. In contrast, Scissor- tumor cells showed a relatively higher expression of PV cell features. CM cells are progenitors of nephrons and can differentiate into PV cells (33). Emerging evidences have shown that cancer cells can acquire the ability to progress or develop drug resistance through onco-fetal reprogramming (51, 52). Therefore, this dedifferentiation of WT tumor cells to a more primitive phenotype may also suggest the existence of an onco-fetal transition in WT recurrence.

To facilitate the clinical application of the Scissor+ tumor phenotype features, we initially employed univariate Cox regression and LASSO analysis to reduce the number of features derived from DEGs of Scissor+ tumor cells, resulting in the generation of the RT-sig comprising 27 genes. Subsequently, an ensemble machine learning model was developed based on this RT-sig to predict RFS in WT patients. This model demonstrated superior performance compared to a combination of clinical characteristics (e.g., sex, age, stage, and histology). Although based on transcriptional features, the risk stratification generated by this ensemble model also reflects adverse or favorable genomic aberrations including mutations in WT-related genes and segmental chromosome CNVs.*TGFA* was identified as the most important gene in the construction of random forest model, consistent with previous studies showing increased expression of *TGFA* in WT is correlated with tumor classification and clinical progression (53). We also validated the elevated expression of TGFA in tumors which further underwent recurrence through immunohistology. However, due to limited sample sizes, a multicenter study with a larger study population is needed to confirm this finding. The establishment of this RFS prognostic model utilizing tumor cell subtypes provides a tailored method for predicting patient outcomes and informing treatment decisions. By accurately identifying high-risk patients, the model demonstrates its potential in patient stratification, enabling more precise treatment approaches such as high-dose therapies or targeted treatments. This personalized strategy could contribute to optimizing therapeutic interventions, ensuring that patients receive the most effective treatment based on the distinct characteristics of their tumors.

The microenvironment of the CSC niche plays an essential role in the formation and maintenance of CSCs. The niche can comprise TME components such as CAFs, immune cells, extracellular matrix, and cytokines, which provide a suitable microenvironment for CSCs, all of which create an optimal microenvironment for CSCs (50, 54). We investigated cellular interactions between Scissor+ tumor cells and other TME cell types. Scissor+ tumor cells have abundant interactions with CAFs. On the one hand, Scissor+ tumor cells secreted ligands that activated IGF2 and PDGF signaling pathways which could regulate the transformation of fibroblasts into CAFs (55, 56). On the other hand, CAFs promote Scissor+ tumor cell survival and invasion through FGF (39, 42, 43) and SLIT2-ROBO (57)signaling pathways. The spatial location of Scissor+ tumor cells was also visualized, where they reside in an immune-desert region, encapsulated by CAFs. This microenvironment may facilitate the evasion of immune cell attack and contribute to immune suppression, thereby supporting tumor survival and progression. Although many studies have already illustrated the interplay between CAF and cancer cells expedites malignant progression (58, 59), more rigorous experiments are needed to validate this finding in WT. Moreover, interrupting connections between CAFs and CSCs through inhibiting key molecules in IGF2, PDGF, SLIT2 signaling pathways could provide potential biomarkers and therapeutic targets for WT.

The rapid advancement of in-silico drug sensitivity prediction, based on bulk or single-cell transcriptomics, has facilitated the identification of potential drugs targeting various tumor phenotypes (30, 60). In this study, by using PERCEPTION pipeline (30), we observed that Scissor+ tumor cells manifested multi-drug resistance to conventional chemotherapy agents including vincristine, doxorubicin, etoposide (1, 61), reinforcing its role as potential CSCs in WT. Notably, though resistant to chemotherapeutics, Scissor+ tumor cells were responsive to targeted therapies, especially EGFR inhibitors (afatinib, icotinib, and osimertinib). EGFR inhibitors have been clinically used to treat malignancies including breast (62), colon (62) and lung cancer (63). Phase I and pharmacokinetic studies of the EGFR inhibitor erlotinib, both as a single agent and in combination with temozolomide, have been conducted in children with refractory solid tumors (64). Our findings suggest EGFR inhibitors could serve as a promising therapeutic option for managing recurrent WT patients; however, further investigation is required to fully elucidate the mechanisms driving the upregulation of *EGFR* in Scissor+ tumor cells. Specifically, the role of key nephron progenitor transcription factors, such as SIX2, in modulating EGFR expression, and whether intervening in this regulatory pathway could inhibit tumor progression and recurrence, warrants deeper exploration. Collectively, these findings indicate that WT patients identified as high-risk for relapse by the ensemble prediction model could benefit from a combined approach that integrates conventional chemotherapy with EGFR-targeted therapies. This strategy has the potential to overcome current treatment limitations and improve outcomes for WT patients.

## 5 Conclusion

In conclusion, seven distinct tumor subgroups with varying expression patterns in WT were characterized based on fetal kidney signatures. By integrating snRNA-seq, spatial transcriptome, and bulk RNA-seq data, we identified a subset of Scissor+ tumor cells associated with poor RFS and tumor recurrence. Scissor+ tumor cells were primarily derived from CM-like-blastemal and fibroblast-like tumor subgroups. These cells exhibit characteristics of cancer stem cells and nephron progenitors, residing in immune-desert niches surrounded by CAFs, and interact through signaling pathways such as *IGF, PDGF*, and *SLIT2*. To facilitate clinical applications, we developed an ensemble machine learning model based on Scissor+ signatures, which not only accurately predicts RFS but also outperforms clinical features and reveals adverse genomic alterations. Elevated expression of *TGFA* was linked to relapse, highlighting its potential as a biomarker. Additionally, Scissor+ cells displayed resistance to conventional chemotherapy agents but sensitivity to EGFR inhibitors. These findings offer valuable insights for personalized treatment strategies aimed at improving outcomes for WT patients at high risk of relapse.

## Data Availability

The datasets presented in this study can be found in online repositories. The names of the repository/repositories and accession number(s) can be found below: https://www.ncbi.nlm.nih.gov/, TARGET; https://www.ncbi.nlm.nih.gov/geo/, GSE31403; https://www.ncbi.nlm.nih.gov/geo/, GSE10320; https://www.ncbi.nlm.nih.gov/geo/, GSE200256.

## Supplementary material

Supplementary Figure 1 Single-nuclear atlas of Wilm tumors from the SCPCP000006 project in the ScPCA portal. **(A)** The UMAP visualization of all cells colored by sample. **(B)** The UMAP visualization of all cells colored by density of tissue collection timepoint. **(C)**The UMAP visualization of all cells colored by density of histology. **(D)** The UMAP visualization of all cells colored by density of relapse status.

Supplementary Figure 2 Heatmap illustrating inferred copy number variations for each cell type.

Supplementary Figure 3 Tumor subgroups recovered from WT snRNA-seq data. **(A, B)** The UMAP visualization of all tumor cells colored by expression of canonical WT epithelial, blastemal and stromal markers. **(C)** The UMAP visualization of all tumor cells colored by WT1 expression. **(D)** The UMAP visualization of all tumor cells colored by normalized expression of REACTOME_REGULATED_NECROSIS. **(E)** The bar plot showing the proportions of tumor subgroups in samples with and without relapse after initial treatment(left). The bar plot showing the proportions of tumor subgroups in unfavorable versus favorable samples(right). **(F)** The bar plot showing the proportions of tumor subgroups across different samples. **(H)** Hematoxylin and eosin (H&E) staining of slide SCPCL000390 (favorable histology) (Left). Spatial distribution of tumor subgroup-specific markers (Right). **(I)** Hematoxylin and eosin (H&E) staining of slide SCPCL000429 (unfavorable histology) (Left). Spatial distribution of tumor subgroup-specific markers (Right).

Supplementary Figure 4 Tumor meta cells generated by pseudobulk analysis. **(A)** The UMAP visualization of meta cells colored by sample. **(B)** The UMAP visualization of meta cells colored by relapse status.

Supplementary Figure 5 Spatial transcriptomic analysis of sample SCPCL000374. **(A)** Spatial distribution of cell type signatures. **(B)** Spatial visualization of inferred signaling pathways in spatial transcriptomics, colored by receiver weight. Arrows indicate the spatial directions of the pathways.

Supplementary Figure 6 Spatial distribution of cell type signatures in representative samples with favorable or anaplastic histology, with and without recurrence.
