# Supplementary figures and images for: Integration of single-nuclei and spatial transcriptomics to decipher tumor phenotype predictive of relapse-free survival in Wilms tumor
Source: Front Immunol. 2025 Mar 3;16:1539897. doi: 10.3389/fimmu.2025.1539897 (PMC11911335; doi:10.3389/fimmu.2025.1539897)

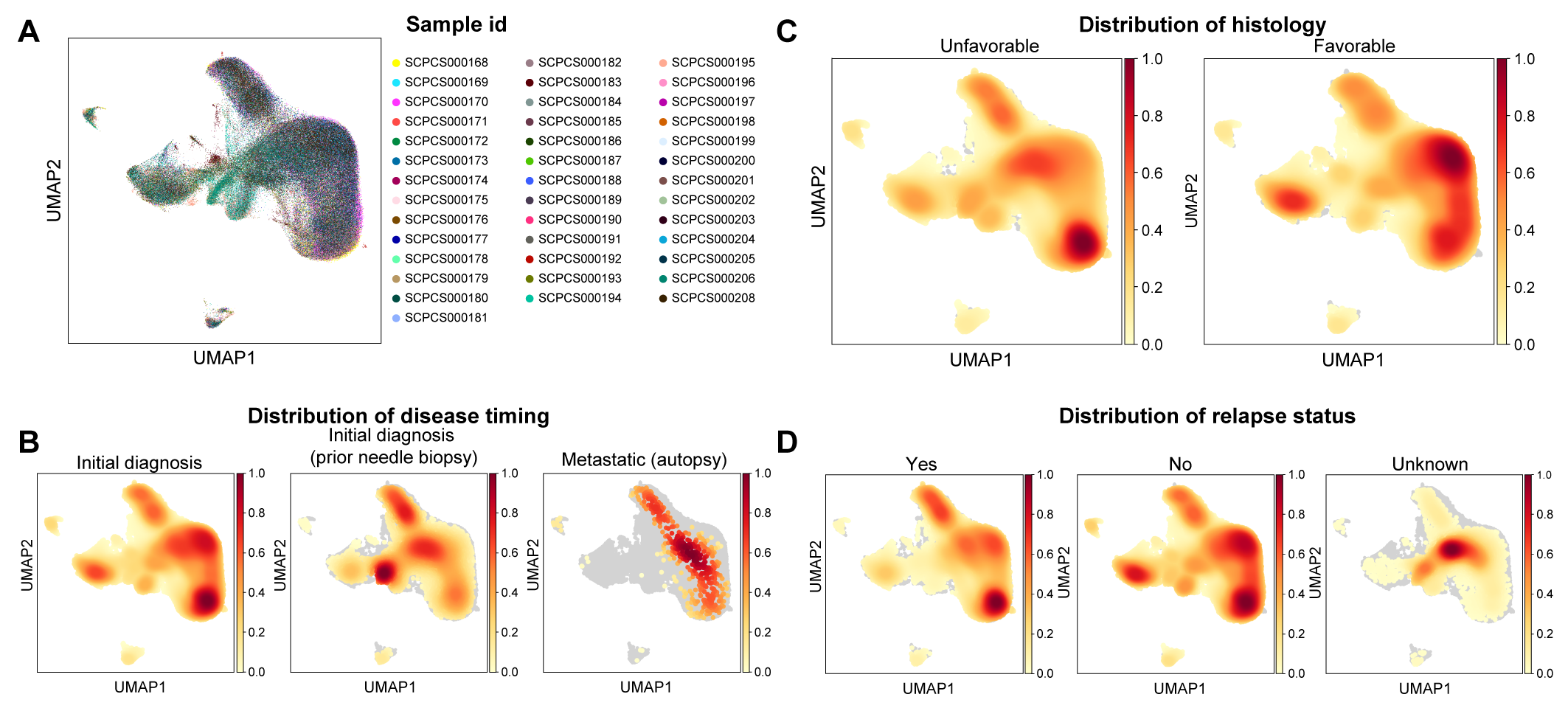

Supplement: Supplementary Figure 1 — Single-nuclear atlas of Wilm tumors from the SCPCP000006 project in the ScPCA portal. (A) The UMAP visualization of all cells colored by sample. (B) The UMAP visualization of all cells colored by density of tissue collection timepoint. (C)The UMAP visualization of all cells colored by density of histology. (D) The UMAP visualization of all cells colored by density of relapse status. [file Image1.tif]

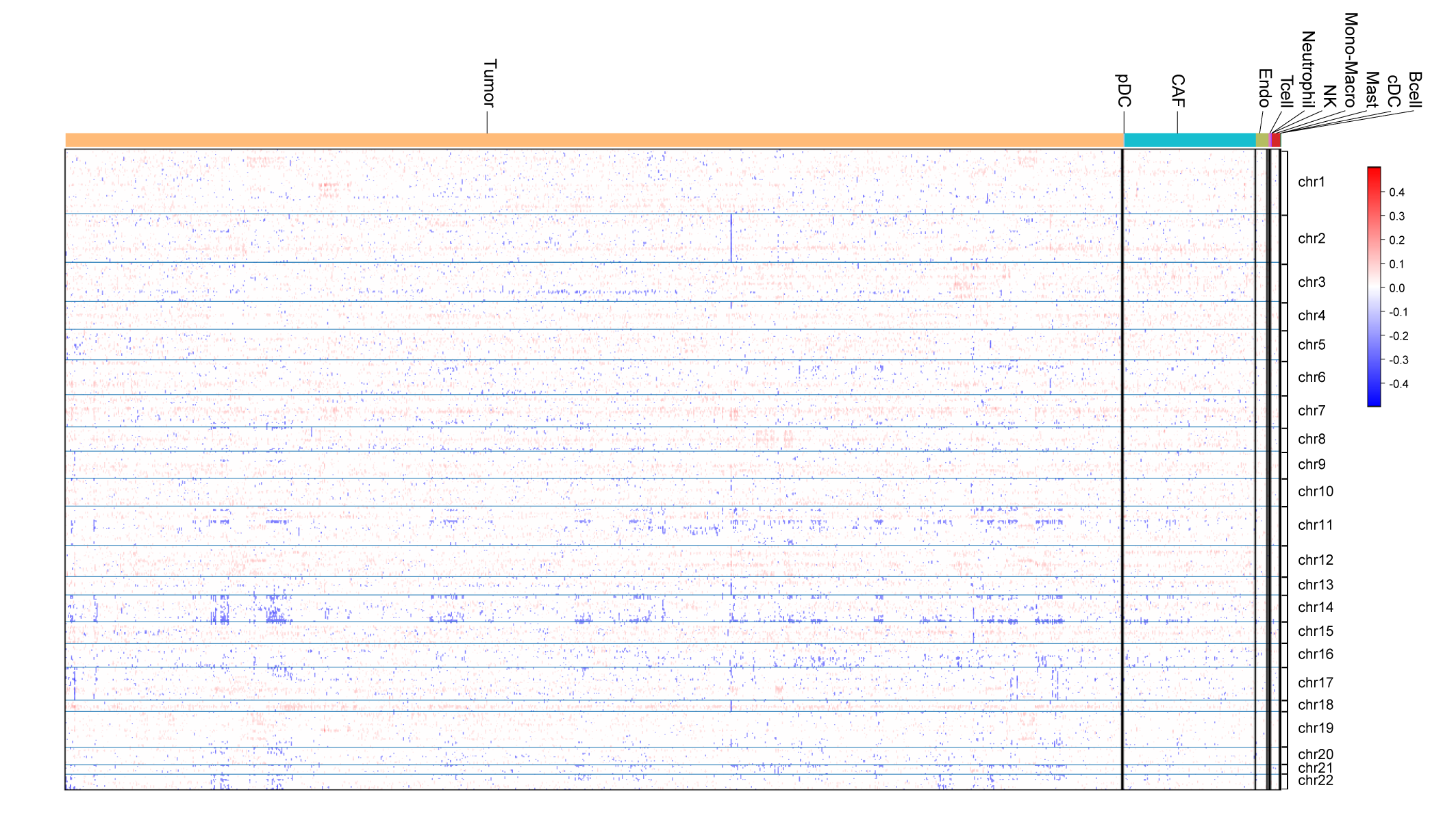

Supplement: Supplementary Figure 2 — Heatmap illustrating inferred copy number variations for each cell type. [file Image2.tif]

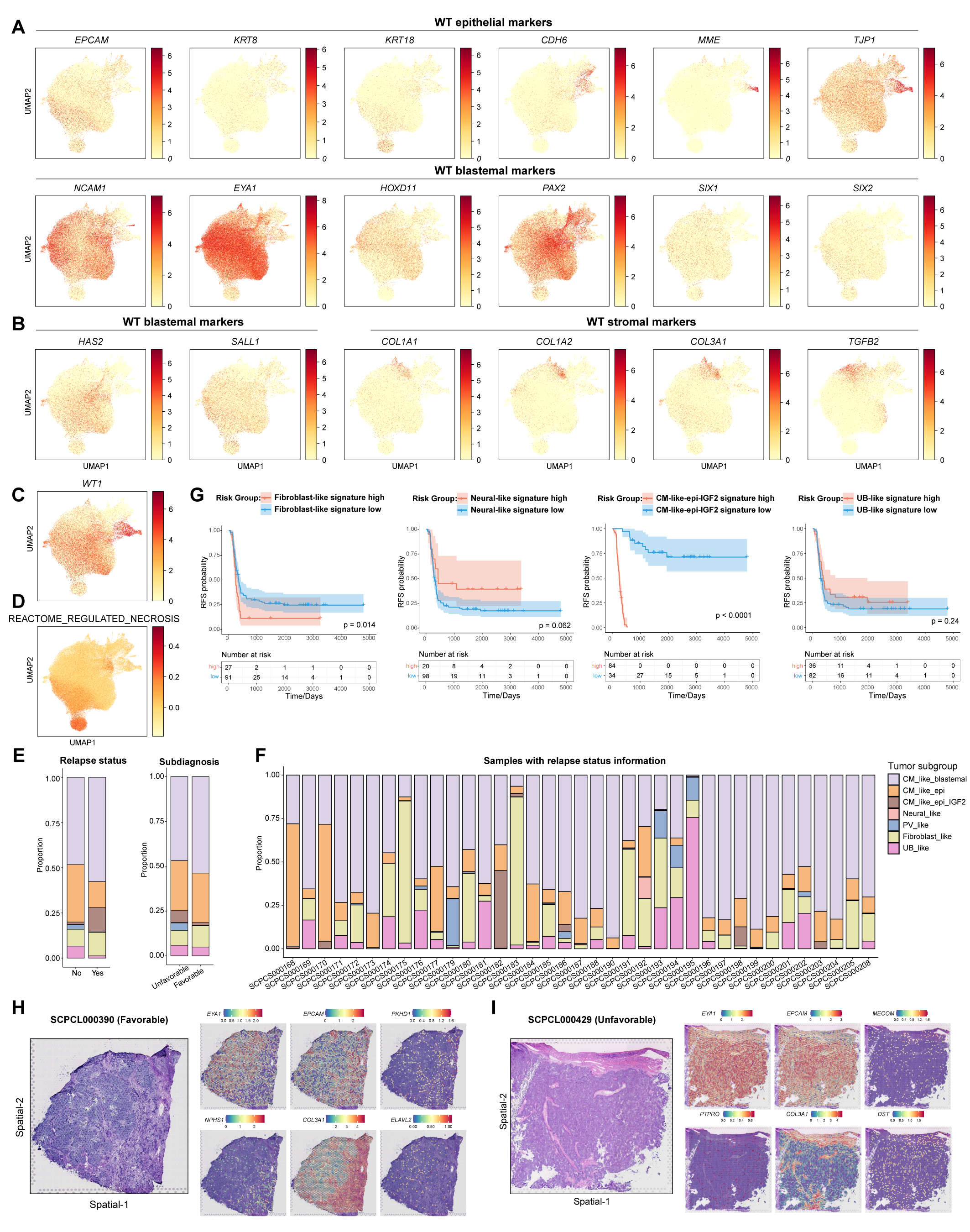

Supplement: Supplementary Figure 3 — Tumor subgroups recovered from WT snRNA-seq data. (A, B) The UMAP visualization of all tumor cells colored by expression of canonical WT epithelial, blastemal and stromal markers. (C) The UMAP visualization of all tumor cells colored by WT1 expression. (D) The UMAP visualization of all tumor cells colored by normalized expression of REACTOME_REGULATED_NECROSIS. (E) The bar plot showing the proportions of tumor subgroups in samples with and without relapse after initial treatment(left). The bar plot showing the proportions of tumor subgroups in unfavorable versus favorable samples(right). (F) The bar plot showing the proportions of tumor subgroups across different samples. (H) Hematoxylin and eosin (H&E) staining of slide SCPCL000390 (favorable histology) (Left). Spatial distribution of tumor subgroup-specific markers (Right). (I) Hematoxylin and eosin (H&E) staining of slide SCPCL000429 (unfavorable histology) (Left). Spatial distribution of tumor subgroup-specific markers (Right). [file Image3.tif]

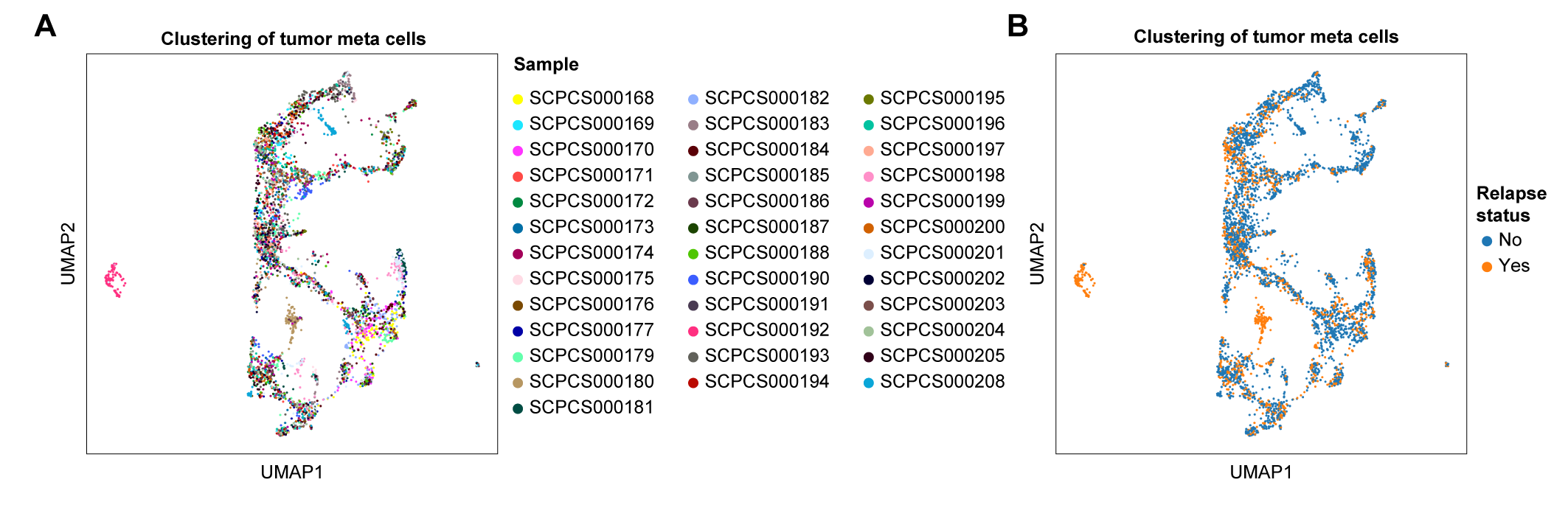

Supplement: Supplementary Figure 4 — Tumor meta cells generated by pseudobulk analysis. (A) The UMAP visualization of meta cells colored by sample. (B) The UMAP visualization of meta cells colored by relapse status. [file Image4.tif]

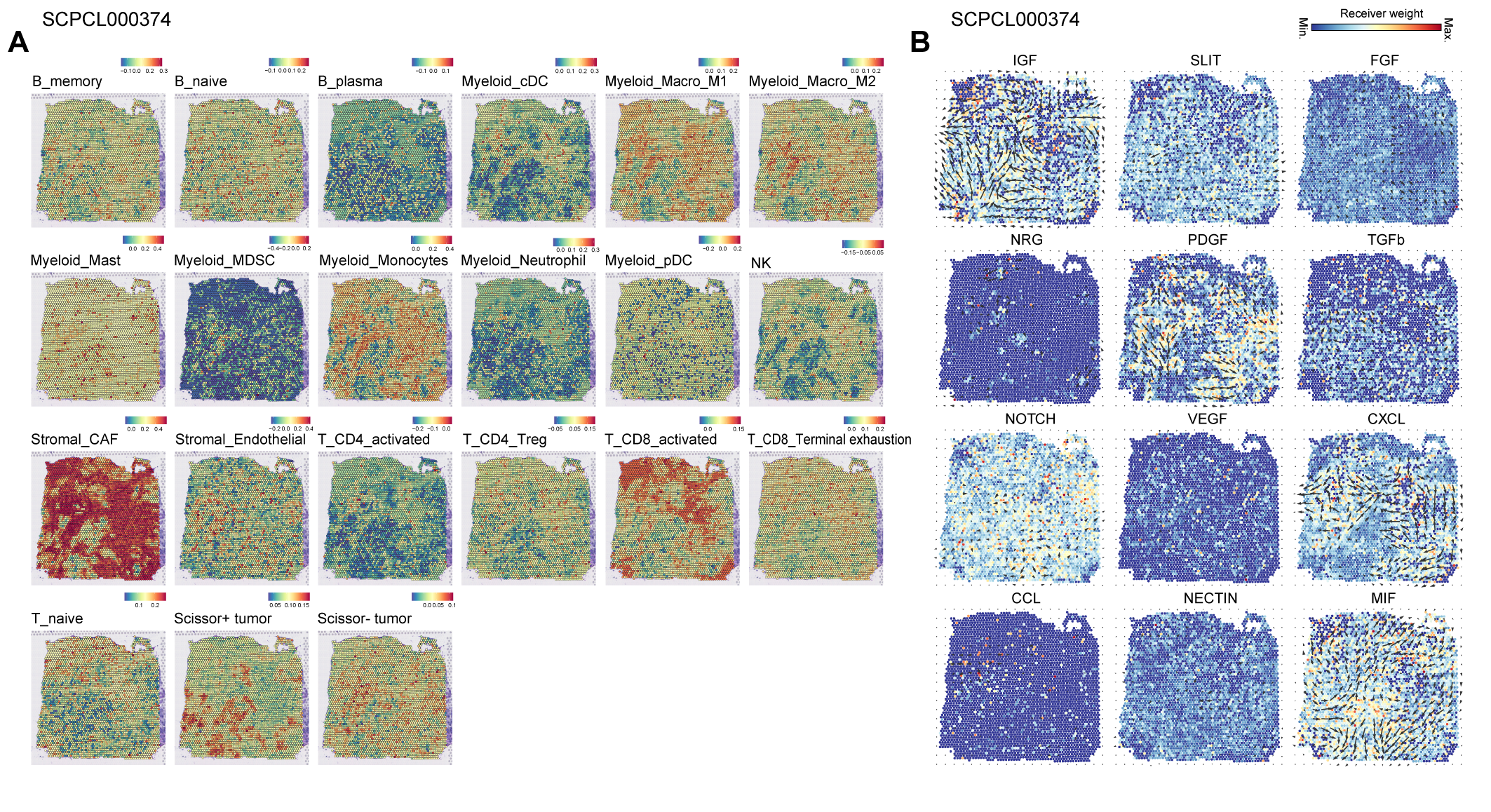

Supplement: Supplementary Figure 5 — Spatial transcriptomic analysis of sample SCPCL000374. (A) Spatial distribution of cell type signatures. (B) Spatial visualization of inferred signaling pathways in spatial transcriptomics, colored by receiver weight. Arrows indicate the spatial directions of the pathways. [file Image5.tif]

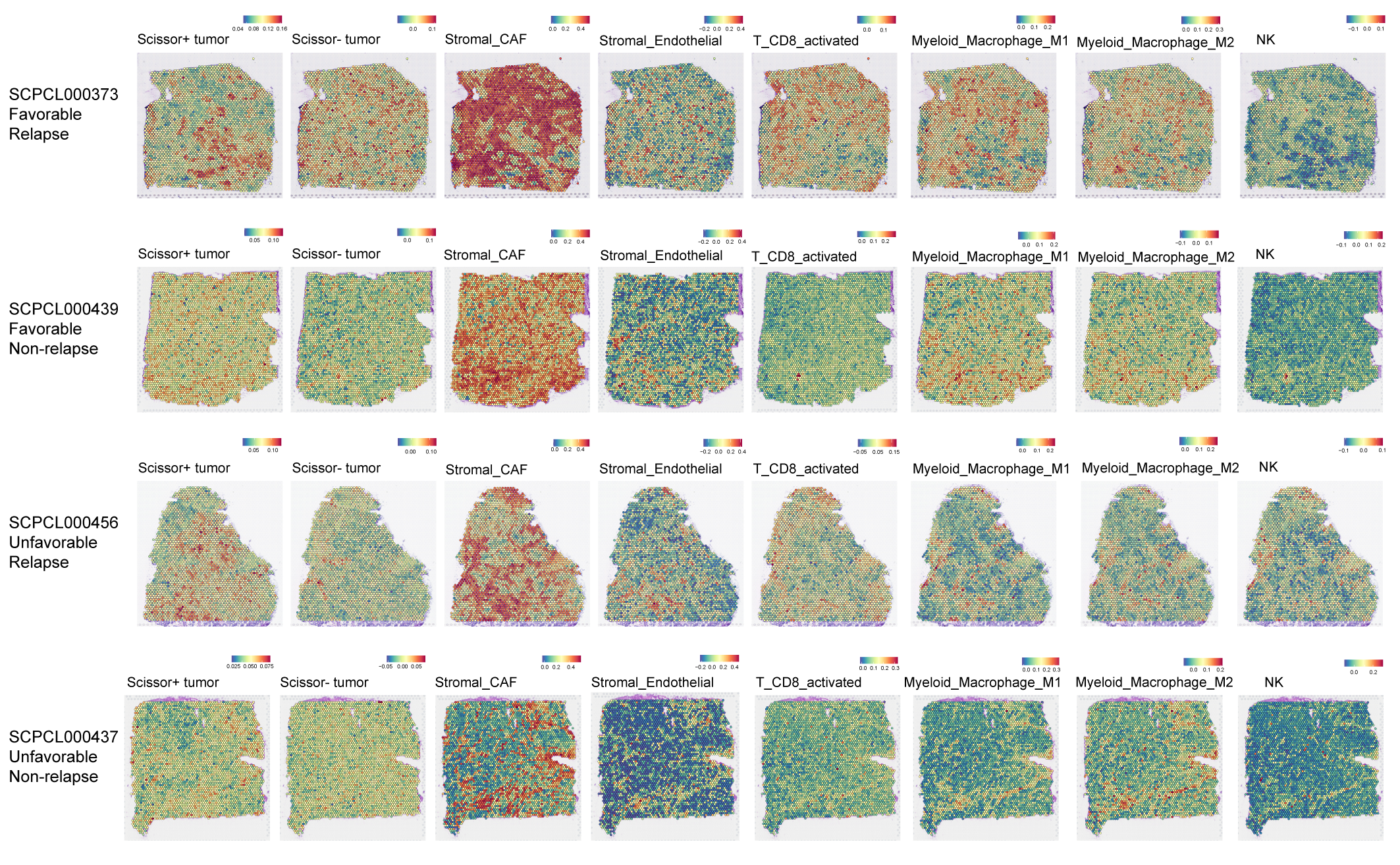

Supplement: Supplementary Figure 6 — Spatial distribution of cell type signatures in representative samples with favorable or anaplastic histology, with and without recurrence. [file Image6.tif]
